# Ultrafast pump-probe phase-randomized tomography

**DOI:** 10.1038/s41377-025-01789-y

**Published:** 2025-03-06

**Authors:** Filippo Glerean, Enrico Maria Rigoni, Giacomo Jarc, Shahla Yasmin Mathengattil, Angela Montanaro, Francesca Giusti, Matteo Mitrano, Fabio Benatti, Daniele Fausti

**Affiliations:** 1 Dipartimento di Fisica, Università degli Studi di Trieste, Trieste, Italy; 2 Sincrotrone Trieste S.C.p.A., Basovizza, Italy; 3 Department of Physics, Harvard University, Cambridge, MA USA; 4 Department of Physics, University of Erlangen-Nürnberg, Erlangen, Germany; 5 Istituto Nazionale di Fisica Nucleare, Sezione di Trieste, Trieste, Italy

**Keywords:** Optical spectroscopy, Optics and photonics, Physical sciences

## Abstract

Measuring fluctuations in matter’s low-energy excitations is the key to unveiling the nature of the non-equilibrium response of materials. A promising outlook in this respect is offered by spectroscopic methods that address matter fluctuations by exploiting the statistical nature of light-matter interactions with weak few-photon probes. Here we report the first implementation of ultrafast phase randomized tomography, combining pump-probe experiments with quantum optical state tomography, to measure the ultrafast non-equilibrium dynamics in complex materials. Our approach utilizes a time-resolved multimode heterodyne detection scheme with phase-randomized coherent ultrashort laser pulses, overcoming the limitations of phase-stable configurations and enabling a robust reconstruction of the statistical distribution of phase-averaged optical observables. This methodology is validated by measuring the coherent phonon response in α-quartz. By tracking the dynamics of the shot-noise limited photon number distribution of few-photon probes with ultrafast resolution, our results set an upper limit to the non-classical features of phononic state in α-quartz and provide a pathway to access non-equilibrium quantum fluctuations in more complex quantum materials.

## Abstract (web summary)

This work leverages the intrinsic carrier-envelope phase instability of pulsed lasers to measure the photon number distribution of ultrashort probes, providing a new way to study quantum fluctuations in light-driven materials.

## Graphical abstract

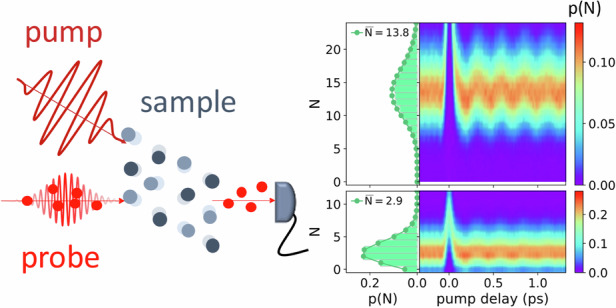

## Introduction

Fluctuations are a fundamental feature of quantum systems and revealing them is a key challenge in understanding some of the most debated exotic states in complex quantum materials^1^ and in designing new quantum devices^2^. Quantum phenomena like superposition, entanglement, and vacuum fluctuations have an inherently statistical nature, which can lead to intriguing macroscopic effects in quantum materials when the quantum correlations survive thermal decoherence. Ultrafast photoexcitation has recently emerged as a powerful means to control and induce new coherent phenomena, like light-induced superconductivity^3–6^, light-induced ferroelectricity^7,8^ and vibrational light-induced transparency^9^, which are otherwise not accessible at the thermodynamic equilibrium. Harnessing these non-equilibrium states requires understanding how the fluctuations of the relevant electronic, vibrational, or magnetic degrees of freedom are modifying the natural thermal evolution of the system.

Treating the light-matter coupling fully at the quantum level, beyond semiclassical approximations, opens new spectroscopic opportunities^10^ to access the fluctuations in materials. The strategy is to investigate the statistical degrees of freedom of matter leveraging on the knowledge of the statistical properties of quantum light developed in quantum optics. The quantum optical properties for instance play a role when considering the ultrafast electron dynamics driven by intense light. Although the strong electric field has always been so far considered classical, the quantum statistical distribution of the light has been proposed to induce the emission of High Harmonic Generation radiation^11–14^ or electrons^15^ with new properties.

**Fig. 1:**
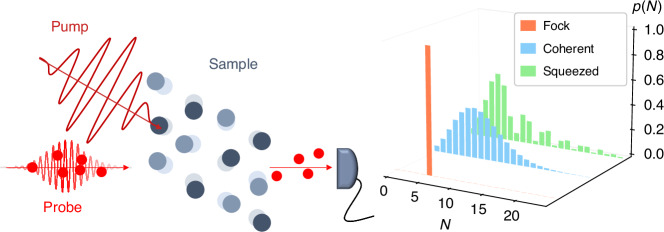
Ultrafast pump-probe spectroscopy of fluctuations measuring the quantum optical statistics of ultrashort laser pulses. We investigate the possibility of accessing the system fluctuations distinguishing the effects that the light-matter interaction produces in the photon number distribution of weak probe pulses, altering the classical coherent state statistics

We explore the quantum character of ultrafast light-matter interaction with a different perspective. Rather than studying the effects of quantum light as input, we investigate how materials can modify the quantum statistical properties of the output light and propose to study the intrinsic quantum fluctuations of the system by imprinting them into the statistical properties of light (Fig. 1). Quantum spectroscopies have been successful in studying quantum fluctuations at equilibrium^16–19^, but their application to ultrafast non-equilibrium phenomena is so far limited to theoretical efforts^10,20^ and a few experimental attempts^21–23^, because it is technically challenging to adapt standard pump-probe experiments to reliably detect the quantum statistical response.

In a pump-probe experiment, a first strong pump pulse impulsively drives the system out of equilibrium and a second probe pulse monitors with femtosecond resolution the relaxation dynamics of the sample excitation. Pump-probe experiments usually detect tiny changes in the average optical intensity of classical probe fields, while the quantum properties of light emerge especially in the weak intensity regime, where the photon discretization comes into play. The statistics of weak light pulses with a few photons per pulse are difficult to measure since direct low-photon counting detection schemes are still at a developing stage^24,25^ and indirect quantum state reconstruction methods rely on delicate and slow phase-resolved interferometric measurements^23,26^.

In this work, we devise an ultrafast quantum spectroscopy method that measures the probe photon number statistics without a phase-stable interferometer, taking advantage of coherent phase-randomized, or phase-averaged (PHAV)^27,28^ states. Weak PHAV states are employed as realistic single-photon sources^29–31^, which are useful to implement decoy states in quantum communication protocols^32^, random number generation^33^, and reveal quantum interference^34^. We develop a phase-randomized heterodyne interferential scheme for measuring the phase-averaged optical quadrature^35,36^, which exploits the intrinsic carrier-envelope phase (CEP) instability of the pulsed laser source. Thanks to phase randomization, we uniformly sample the optical phase space of the probe field and obtain the technical advantage of not being affected by phase stability issues. It is not necessary to measure the phase-resolved mean-value oscillation of the optical quadrature field, but we collect the phase-averaged quadrature distribution and reconstruct with tomography the full photon number distribution of the probe state. We highlight that, since the natural CEP fluctuations are perfectly uniformly distributed and uncorrelated, our method is more reliable and efficient than any phase manipulation protocol (see Supplementary Material for detailed characterization and discussion).

## Results

### Phase-randomized ultrafast optical tomography

**Fig. 2:**
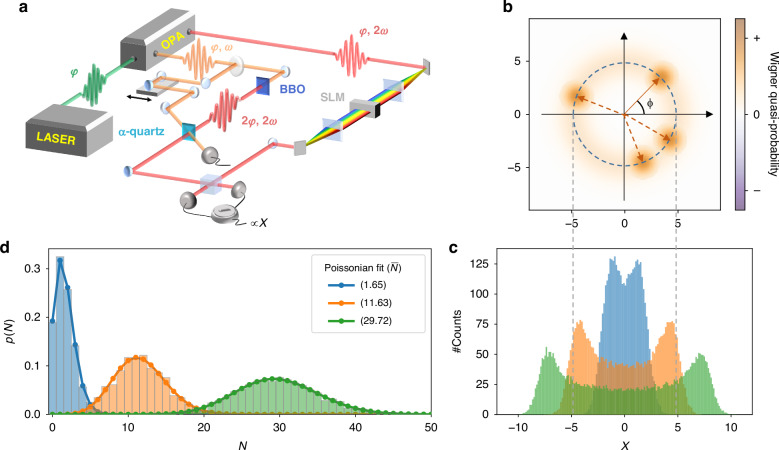
Reconstruction of the photon number distribution with a phase-randomized pump-probe experiment. **a** Experimental scheme. The signal and idler outputs of an optical parametric amplifier (OPA), such that ω_SIG_ = 2ω_IDL_, are used in combination with a second harmonic generation process to set up a phase-averaged pump-probe heterodyne detection sensitive to the random laser CEP (details in text). **b** Wigner distribution of the phase-averaged coherent state resulting from the randomization of the CEP-dependent LO-probe phase. **c** The detection output is the distribution of the phase-averaged quadrature. **d** Applying the tomography procedure to the quadrature data we obtain the probe photon number distribution

The experimental setup (represented in Fig. 2a) stems from the combination of a pump-probe scheme with optical state tomography. It is an evolution of a multimode heterodyne interferometer^26^, optimized for the study of phase-averaged observables of the weak optical probe. The ultrashort pulses provided by the laser source are coherent states. The measurement of the quantum statistics of weak coherent states relies on the continuous variable analysis performed through optical tomography^37^. The coherent state is represented in the optical phase space (Fig. 2b) as a minimum uncertainty Wigner distribution, characterized by amplitude, *α*, and phase, *φ*, in analogy with a classical field. The Wigner function *W* is associated with the quantum optical state and allows us to predict the mean values of the generic observable *O* with integration over the full phase-space

1 $${{\langle }}O{{\rangle }}=2\pi {{\hslash }}\int \int {dXdY\; W}(X,\,Y)\,\widetilde{O}(X,\,Y)$$

where *X* and *Y* are the two phase-space quadratures and $\widetilde{O}$ the Wigner–Weyl transform of the observable *O*. The Wigner–Weyl transform of the operator *O* is defined as $\widetilde{O}=1/2\pi \hslash \int {dx}\left\langle X+x/2\left|O\right|X-x/2\right\rangle {e}^{{ixY}/\hslash }$. The Wigner function *W* is the Wigner–Weyl transform of the density operator that describes the quantum state. The Wigner distribution can be reconstructed through the tomography algorithm by measuring the generalized quadrature for different phase projections in the optical phase space as

2 $$X\phi =\frac{1}{\sqrt{2}}(a{e}^{i\phi }+{a}^{\dagger }{e}^{-i\phi })$$

where *a* and *a*^†^ are the ladder operators related to the quantized optical mode. The quadrature is usually measured with a homodyne detection setup, where the intense classical local oscillator (LO) field amplifies the weak quantum optical probe and their interference is detected with a balanced detection scheme. The projection phase is referenced as the relative phase *ϕ* between the probe and the LO beam, which in a conventional phase-stable interferometer is controlled by modifying the optical delay between the two. To study phase-averaged observables of a coherent state, such as the photon number distribution, we do not need to resolve the phase-dependent field profile, but we can alternatively measure the statistical distribution of the phase-integrated quadrature^35,36,38^ (Fig. 2c). In detail, we employ phase-averaged coherent states which have a ring-like Wigner distribution in the optical phase-space as a result of the integration around all the possible phases (Fig. 2b)

3 $${X}_{{\rm{PHAV}}}=\frac{1}{2\pi }{\int }_{0}^{2\pi }d\phi \frac{1}{\sqrt 2}\left(a{e}^{i\phi }+{a}^{\dagger }{e}^{-i\phi }\right)$$

which has a distribution whose width depends on the photon number (Fig. 2c). Starting from the PHAV quadrature distribution, we use a maximum likelihood algorithm^39–41^ to calculate the photon number distribution on the Fock space (details in the Supplementary). We show in Fig. 2d the agreement between the Poissonian shape and the reconstructed data for probes with different mean values of photons per pulse. We report that the numerical limitations of the algorithm allow us to calculate the distribution of up to 150 photons (see Supplementary).

The present discussion is limited to a single frequency component of the photon field. Nevertheless, we note that the ultrashort pulses are multimode. We underline that by exploiting the shaping of the LO spectrum we can frequency-resolve the probe response^26^ (characterization of the multimode equilibrium spectrum is reported in the Supplementary). Here, we show only the results relative to a narrow frequency band at the center of the probe spectrum, which is representative of the time-dependent response observed in all the spectral bandwidth^42^.

We generate the PHAV probe pulses for our experiment exploiting the random CEP^43^ of ultrashort laser pulses. In a conventional homodyne scheme, the probe and LO are split from the same laser beam, and the random CEP phase is conveniently canceled out in the interference between the two. Importantly, we instead preserve the CEP fluctuation to measure a uniformly distributed set of quadrature phases, without the need to resolve the quadrature phase with a stable interferometer. To achieve sensitivity to the random CEP phase, *φ*_*CEP*_, we take advantage of the Second Harmonic (SH) generation process, which is widely exploited to implement CEP control systems^44^. As depicted in Fig. 2a, the signal and idler outputs of an optical parametric amplifier (OPA) are tuned such that the SH of the idler is resonant with the signal. The idler beam is split in two: one portion is used as a pump and routed through a delay stage, while the other generates the probe beam with an SH generation process. The relative CEP between the two OPA outputs is the same because both the seed white-light generation and the amplification stages are pumped by the SH of the laser fundamental^45^. The probe is generated as SH of the idler beam on a BBO crystal. Prior to the interaction with the sample, bandpass filters remove the idler fundamental, and neutral filters attenuate the probe to the few-photon regime. The OPA signal beam is employed as LO. The SH process doubles the CEP and the relative phase between the probe and LO results

4 $$\phi ={\varphi }_{{probe}}-{\varphi }_{{LO}}={2\varphi }_{{CEP}}-{\varphi }_{{CEP}}={\varphi }_{{CEP}}$$

which makes the quadrature phase *ϕ* randomized, as the initial laser CEP *φ*_*CEP*_.

### Phonon-dependent photon number distribution

We apply the pump-probe PHAV tomography to the study of the non-equilibrium statistical properties of materials. We benchmark the methodology studying the coherent phonon excitation by means of Impulsive Stimulated Raman Scattering (ISRS)^42,46,47^ in α-quartz^42,48,49^, which is a prototypical example of interaction between electromagnetic fields and vibrational states in matter. A proper selection of the polarization of the pump and probe allows for the selection of the response associated with a phonon mode of specific symmetry (the *E*-symmetry mode at 4 THz)^50^. We orient the pump at +45° with respect to the probe. We study the probe scattering in the weak residual polarization, which we select with an analyzer orthogonal to the main probe polarization axis. The response is the result of a non-linear Raman interaction with the photons in the parallel polarization^42^, which results in the scattering of photons between the two polarization components and modulates the ellipticity of the transmitted light.

**Fig. 3:**
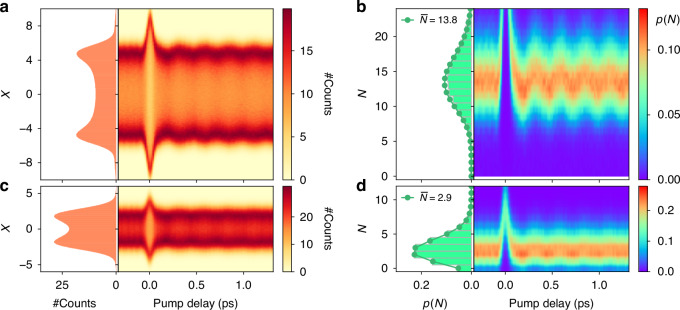
Pump-probe modulation of the photon number distribution induced by the coherent phonon excitation. **a** Phase-averaged quadrature distribution for a probe pulse with a mean photon number of 13.8 photons per pulse. Left: histogram of the equilibrium phase-averaged quadrature distribution. Right: histogram map describing the time-resolved dynamics of the quadrature distribution. **b** Applying the tomography algorithm to the quadrature data we study the evolution of the photon number distribution. Equilibrium (left) and time-resolved evolution (right) are shown. We also consider a weaker probe beam with an average of 2.9 photons per pulse and report the relative evolution for the quadrature (**c**) and photon number (**d**) distributions. We observe in both cases the presence of coherent oscillations at the phonon frequency

The direct output of the detection system is the quadrature distribution. In Fig. 3a, c we see the equilibrium quadrature and its non-equilibrium modulation for two different intensities of the weak probe state. As a function of the time delay, we see that the quadrature distribution changes its width. At the zero delay, we have a strong response due to the coherent overlap with the pump pulse, but more importantly, we see oscillations at positive times. The latter is due to intensity, i.e., average photon number, and changes in the orthogonal polarization induced by the phonon excitation. Applying the phase-averaged tomography to the quadrature distribution data, we can study the non-equilibrium response of the photon number distribution. The equilibrium distribution in Fig. 3b, d (left) is well fitted by a Poissonian distribution with an average photon number of $\bar{N}$ = 13.8 and 2.9 photons per pulse, respectively. We observe in Fig. 3b, d (right) that the probability distribution is shifting following the phonon oscillations.

In order to understand how the interaction of the probe with the phonon mode modifies its photon number distribution, we model the light-phonon interaction with a Raman Hamiltonian^42^

5 $$\begin{array}{l}{H}_{{Ram}}=-\mathop{\sum }\limits_{\omega }\chi \left({a}_{x,\omega +\varOmega }^{\dagger }{a}_{y,\omega }b+{a}_{x,\omega +\varOmega }{a}_{y,\omega }^{\dagger }{b}^{\dagger }\right.\\\qquad\quad+\,\left.{a}_{y,\omega +\varOmega }^{\dagger }{a}_{x,\omega }b+{a}_{y,\omega +\varOmega }{a}_{x,\omega }^{\dagger }{b}^{\dagger }\right)\end{array}$$

which describes the scattering between the optical polarizations *x* and *y* mediated by $\chi ={\chi }_{{xy}}$, the non-linear polarizability tensor associated with the *E*_*T*_ phonon. The operator *b* is the field of the phonon with frequency Ω, while the photon frequency ω runs over the spectrum of the light pulse. In the specific polarization configuration considered, the pump-probe response is spectrally uniform^42^, and for simplicity, we will neglect the frequency index *ω*. We define *x* as the main polarization axis and *y* as the weak cross-polarized residual. The input optical coherent states are defined as ${a}_{x}|{\alpha }_{x}\rangle ={\alpha }_{x}|{\alpha }_{x}\rangle$, ${a}_{y}|{\alpha }_{y}\rangle ={|\alpha }_{y}|{e}^{-i\pi /2}|{\alpha }_{y}\rangle$, where the factor *π*/2 accounts for the phase shift between the two polarization components due to ellipticity.

We are interested in calculating the response of the experimental observable, i.e., the photon number operator $N={a}_{y}^{\dagger }{a}_{y}$ of the weak *y* polarization. Using a perturbative expansion (with $\tau \chi < < 1$, where *τ* is the interaction duration) we can study the phonon-dependent optical response. We calculate (full derivation in the Supplementary) the average photon number as a function of the pump-probe delay Δ*t*

6 $$\bar{N}\left(\Delta t\right)={\alpha }_{y}^{2}+2\tau \chi {(|\alpha }_{x}{||}{\alpha }_{y}|)\left\langle{b}_{\varDelta t}^{\dagger }+{b}_{\Delta t}\right\rangle$$

and its variance.

7 $$\begin{array}{lll}{\sigma }_{N}^{2}\left(\varDelta t\right) & = & \bar{N}\left(\varDelta t\right)\\ && +\,4{\tau }^{2}{\chi }^{2}{\alpha }_{y}^{2}{\alpha }_{x}^{2}\left(\left\langle {{b}_{\varDelta t}^{\dagger }}^{2}\right\rangle -{\left\langle {b}_{\varDelta t}^{\dagger }\right\rangle }^{2}+2\left(\left\langle {b}^{\dagger }{b}_{\varDelta t}\right\rangle\right.\right. \\ && \left.\left.-\left\langle {b}_{\varDelta t}^{\dagger }\right\rangle \left\langle {b}_{\varDelta t}\right\rangle \right)+\left\langle {b}_{\varDelta t}^{2}\right\rangle -{\left\langle {b}_{\varDelta t}\right\rangle }^{2}+1\right)\\ && +\,{\tau }^{2}{\chi }^{2}{\alpha }_{y}^{2}\left(2(\left\langle {b}^{\dagger }{b}_{\varDelta t}\right\rangle -\left\langle {b}_{\varDelta t}^{\dagger }\right\rangle \left\langle {b}_{\varDelta t}\right\rangle )+1\right)\end{array}$$

**Fig. 4:**
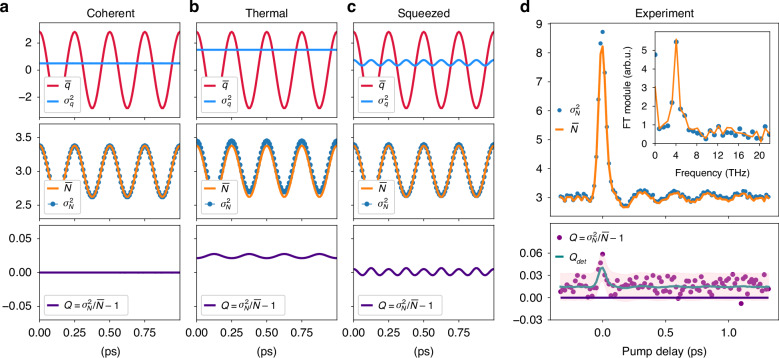
Phonon-dependent evolution of the photon distribution parameters. **a**–**c** We simulate the optical response for coherent (**a**), thermal (**b**), and squeezed (**c**) phonon states. The top panel compares the average phonon displacement and its variance. The middle panel reports the optical response (average and variance of the photon number). The Mandel parameter *Q* (bottom) quantifies the deviations from the coherent state Poissonian statistics. **d** The experimental mean photon number and variance oscillate at the 4 THz phonon frequency, as shown by the Fourier Transform analysis of the positive times (insert). The data are consistent with the detector response *Q*_det_ (green line), which describes a Poissonian behavior (*Q* = 0) corrected considering the intensity-dependent classical excess noise (see Supplementary). The pink area accounts for the error calculated as the standard deviation of repeated measurements of *Q* (2σ)

The mean value oscillates around the initial value at the phonon frequency, ruled by the phonon displacement *q* ∝ *b* + *b*^†^. The variance depends on second-order terms of the phonon operator, which means it is in principle sensitive to the phonon statistics. To understand how the variance of the photon number changes according to the phonon properties, we simulate^51^ it for different phonon states. We present in Fig. 4 the expected results for a displaced phonon state with coherent (a), thermal (b), and squeezed (c) statistics. We consider the phonon field as a large ensemble of oscillators each with a relatively large excitation amplitude |〈*b*〉| = 2 (corresponding to a temperature increase of ~900 K) and set the cross-section (*τχ*) to match the experimentally observed photon number modulation (details on the dependence of the parameters in the Supplementary). The variance of the phonon displacement shows different noise levels and periodicities, peculiar to each phonon state, which are mapped in the optical degrees of freedom by the probe-phonon interaction. We characterize the resulting probe photon distributions in terms of the Mandel parameter *Q*,

8 $$Q=\frac{{\sigma }_{N}^{2}-\bar{N}}{\bar{N}}$$

which quantifies the deviations from Poissonian photon statistics as a function of the difference between mean ($\bar{N}$) and variance (${\sigma }_{N}^{2}$). The Mandel parameter is related to the second-order correlation function $Q=\bar{N}({g}^{2}(0)-1)$. For an optical coherent state, we expect a Poissonian distribution with *Q* = 0, with (i.e., $\bar{N}={\sigma }_{N}^{2}$), while *Q* > 0 or *Q* < 0 describes respectively a super- or sub-Poissonian statistics. The thermal state generates an optical super-Poissonian distribution oscillating at the phonon frequency. The squeezed state produces instead an oscillation at 2Ω around *Q* = 0, with its phase determining the shift between the variance modulation and the phonon wave.

To discuss the capabilities and limitations of the proposed technique, we compare the model and experiment presenting in Fig. 4d the measured evolution of the photon number mean-value and variance for the $\bar{N}$ = 2.9 probe, extracted from the data in Fig. 3d. We are able to resolve that both the average photon number and the variance oscillate at the phonon frequency. If we consider the pump-probe response of *Q*, we observe a small super-Poissonian character (*Q* > 0), which is due to classical experimental excess noise. If we correct the Mandel parameter considering the excess noise which affects the detection response, and define *Q*_det_ (see Supplementary), we can explain the deviation from the equilibrium behavior.

## Discussion

The experiment and the calculations performed in this work validate a new methodology to perform ultrafast time-resolved quantum spectroscopy experiments, which is suitable to reveal signatures of the quantum nature of light-matter interaction in the phase-averaged optical statistical degrees of freedom. We proved the capability to measure non-equilibrium changes in the photon number distribution with ultrafast resolution and we showed theoretically that the material fluctuations can qualitatively perturb the optical statistics. Our measurement on quartz sets a bound on the amount of squeezing or thermal excitations present in the system.

The model predicts qualitative changes in the optical response as a function of the phonon fluctuations, which are quantitatively comparable to the detection noise. Using a Bose-Einstein distribution $\left(n=1/({e}^{\hslash \Omega /{k}_{B}T}-1)\right)$ we can expect an equilibrium average occupation of 0.7 levels for the 4 THz (16.5 meV) phonon at 300 K. The state reported in Fig. 4b is simulated with a thermal occupation of 1, which should be observable with the current experimental conditions. The result suggests that the non-equilibrium state is well-described by a coherent excitation, without an appreciable injection of incoherent thermal population. To reveal possible modifications due to the quantum fluctuations in this system a higher acquisition statistics is required. This issue can be overcome using a laser source with a higher repetition rate. The current setup could successfully reveal quantum effects in systems with a more pronounced non-Poissonian character or stronger light-matter couplings.

This method establishes a direct connection between the quantum fluctuations of a material and the statistical properties of the electric fields, thus potentially constituting an indicator of “quantumness”. Since the variance of the photon number encodes the variance of microscopic observables, this quantity could be used as a tool to witness entanglement in many-body systems of interest. The variance of a quantum operator is the sum of a thermal/incoherent part that can be separated from the coherent/quantum part, the quantum variance^52^, which provides a lower bound for fundamental quantum estimators, such as the quantum Fisher information (QFI). The QFI is a witness of multipartite entanglement^53^, which can be quantified in solids by performing a full integration over the energy spectrum of mean-value dynamical susceptibilities^54,55^. Our method constitutes a direct statistical approach to probing quantum fluctuations of a macroscopic solid and to bound the entanglement associated with specific degrees of freedom.

In addition to quantum states, the method can allow us to explore statistical effects also in the classical regime. Unlike the uniform coherent phonon excitation in quartz, strong fluctuations can be present in an inhomogeneous system, where the state is a statistical mixture of distinguishable oscillators. For instance, if the dephasing between different oscillators is faster than the population decay, we can expect the evolution of the system into an incoherent state with super-Poissonian statistics. The classical statistical response can then be used in this framework to distinguish the response of the dynamics of population and coherence^56^.

In perspective, the proposed method opens the way to a new typology of quantum spectroscopy based on the study of the statistical response of weak coherent laser pulses, which can be useful to design ultrafast photonic quantum devices and reveal insight into the non-equilibrium dynamics of fluctuations in complex materials.

## Materials and methods

The sample is an α-quartz crystal, with a 1 mm thickness.

The laser pulses are obtained from a pulsed source+OPA system (Pharos + Orpheus, LightConversion). The laser source delivers 1026 nm, 120 fs pulses at 1 kHz repetition rate and we set the outputs of the OPA (signal and idler) such that the SH of the idler (1540 nm, 0.805 eV, <100 fs) is resonant to the signal (770 nm, 1.61 eV, <50 fs).

The experiment is performed in transmission. The equilibrium ratio between parallel and residual intensities due to quartz birefringence is about 100. The fluences on the quartz sample are 4 mJ/cm^2^ for the pump and 2–8 nJ/cm^2^ for the probe. The employed LO has parallel polarization with respect to the detected probe and the full intensity at the detection beam-splitter is about 1 pJ (10^9^ photons/pulse). The reported data are acquired with a spectrally shaped LO with a narrow 0.5 meV bandwidth, centered at 1.62 eV (10^7^ photons/pulse). We collect trains of 4000 pulses per delay point and scan the pump-probe trace 60 times.

The phonon statistics and evolution are simulated using the software tools from the QuTiP package^51^.

## Supplementary information

Supplementary Information
