# Supplementary material for: Ultrafast pump-probe phase-randomized tomography
Source: Light Sci Appl. 2025 Mar 6;14:115. doi: 10.1038/s41377-025-01789-y (PMC11882910; doi:10.1038/s41377-025-01789-y)
Supplement: Supplementary file 1 — Supplementary Information [file 41377_2025_1789_MOESM1_ESM.pdf]

Supplementary Information for

Ultrafast pump-probe phase-randomized tomography

Filippo Glerean[1,2,3], Enrico Maria Rigoni[1,2], Giacomo Jarc[1,2,4],
Shahla Yasmin Mathengattil[1,2], Angela Montanaro[1,2,4], Francesca Giusti[1,2],
Matteo Mitrano[3], Fabio Benatti[1,2,5], and Daniele Fausti[1,2,4,*]

[1] *Dipartimento di Fisica, Università degli Studi di Trieste, Trieste I-34127, Italy*
[2] *Sincrotrone Trieste S.C.p.A., Basovizza I-34149, Italy*
[3] *Department of Physics, Harvard University, Cambridge, MA-02138, USA*
[4] *Department of Physics, University of Erlangen-Nürnberg, 91058 Erlangen, Germany*
[5] *Istituto Nazionale di Fisica Nucleare, Sezione di Trieste, Trieste I-34014, Italy*
[*] daniele.fausti@fau.de

Supplementary materials

A. Uniform random sampling of the quadrature phase

B. Multimode probe characterization

C. Quantum shot-noise characterization

D. Quantum model for Impulsive Stimulated Raman Scattering

E. Statistical properties of the phonon state

F. Maximum likelihood phase-averaged tomography algorithm

A Uniform random sampling of the quadrature phase

We study the randomness of the Carrier-Envelope Phase (CEP) statistical distribution and its key role in ensuring a reliable measurement of the phase-averaged quadrature distribution of the optical probe state. The intrinsic CEP fluctuations exploited in our technique are perfectly uniformly distributed and uncorrelated, granting a homogeneous sampling of the phase of the quadrature which is insensitive to the phase instabilities affecting the interferometer.
We demonstrate the randomness of the CEP adopted in our experiment by analyzing the quadrature values recorded individually for a train of consecutive probe pulses. In Fig.S1a, we observe that the measurements of the optical quadrature, $X = \frac{1}{\sqrt{2}}(ae^{i\phi} + a^\dagger e^{-i\phi})$, span the entire amplitude range without any distinguishable trend between subsequent pulses. We note that the increased occurrence of measurements at the extremes of the range of X is consistent with the shape of the quadrature distributions presented in the main text (Fig.2c), which is a consequence of the projection of the ring-like Wigner distribution of a phase-averaged coherent state. The features of the measured dataset are consistent with those of a simulated dataset (Fig.S1b), where we assume a uniform random distribution for ϕ between 0 and 2π, and add a Gaussian fluctuation δ ($\sigma_\delta^2 = 1/2$) to the quadrature to account for the vacuum fluctuations. The agreement between experiment and simulation also holds if we analyze the correlation between successive pulses by plotting the value recorded for the $(i+1)^{\text{th}}$ pulse as a function of the preceding one. As shown in Fig.S1c,d, no trend is revealed, meaning that correlations between successive pulses are absent. In Fig.S1e, we quantitatively verify the absence of correlations in a train of subsequent measurements calculating the Pearson correlation coefficient as a function of the distance between pulses, which trivially equals 1 for the auto-correlation and reads zero for any other following pulse.

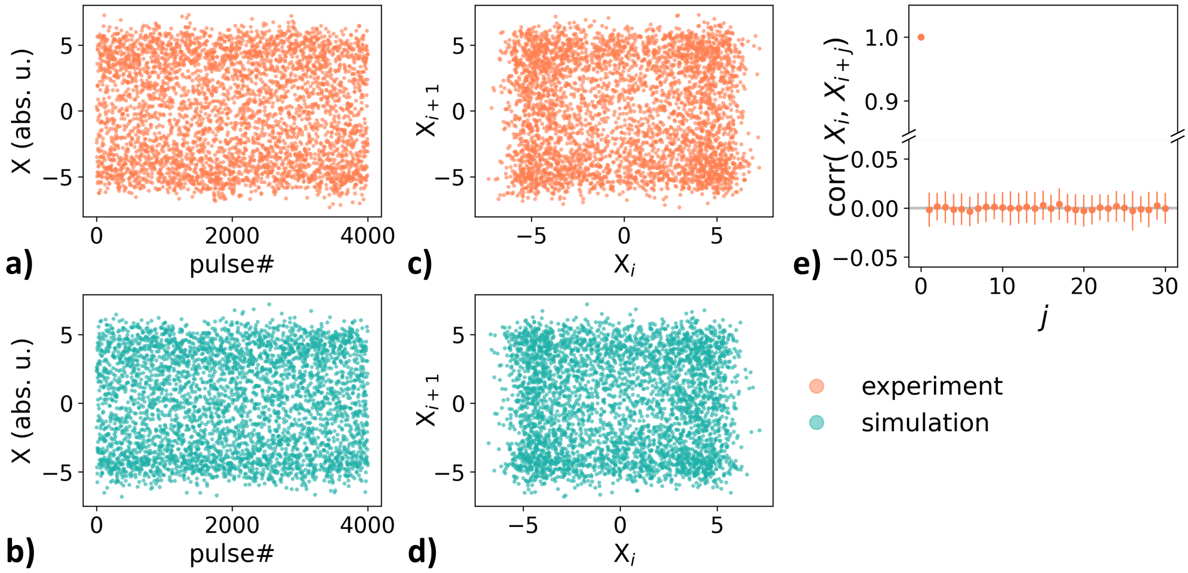

Figure S1: Characterization of Carrier-Envelope Phase randomness, comparing experimental data with simulations using a uniformly random phase. **(a)** Experimental and **(b)** simulated quadrature values for consecutive single-pulse acquisitions. **(c, d)** Lack of correlation between individual quadrature measurements of two adjacent pulses. **(e)** Correlation coefficient calculated between pairs of quadrature acquisitions in a sequence of subsequent pulses.

Conducting phase-averaged studies using the uniform and uncorrelated phase sampling resulting from CEP instability is the most reliable and time-efficient approach. In Fig.S2, we report simulated results comparing the phase-randomized method with other schemes that use a controllable phase (for instance, piezoelectric translators to finely tune the interferometer path

length). The random CEP scheme has three main advantages: i) the phases are sampled in a perfectly uniform way, ii) the time to switch between different phases is as fast as the repetition rate of the employed pulses, and iii) it is insensitive to environmental instabilities which can produce both slow drifts and sudden jumps in the phase recorded by the interferometer. A controlled sampling of a reduced number of phases (Fig.S2b) would introduce artifacts in the quadrature distribution due to finite sampling and would be slowed down by the dead times spent tuning the phase during the acquisition scans. A continuously varying linear phase scan (Fig.S2c) would be a better strategy to generate a correct phase-averaged quadrature distribution, but any systematic deviation from perfect linearity (d) or stochastic fluctuation (e) would distort the final result. Thus, phase-controlled methods can be considered as alternatives for implementing approximately exact phase-averaged tomography, but the ability to exploit the properties of CEP instability makes the phase-randomized method the optimal choice for phase-averaged protocols.

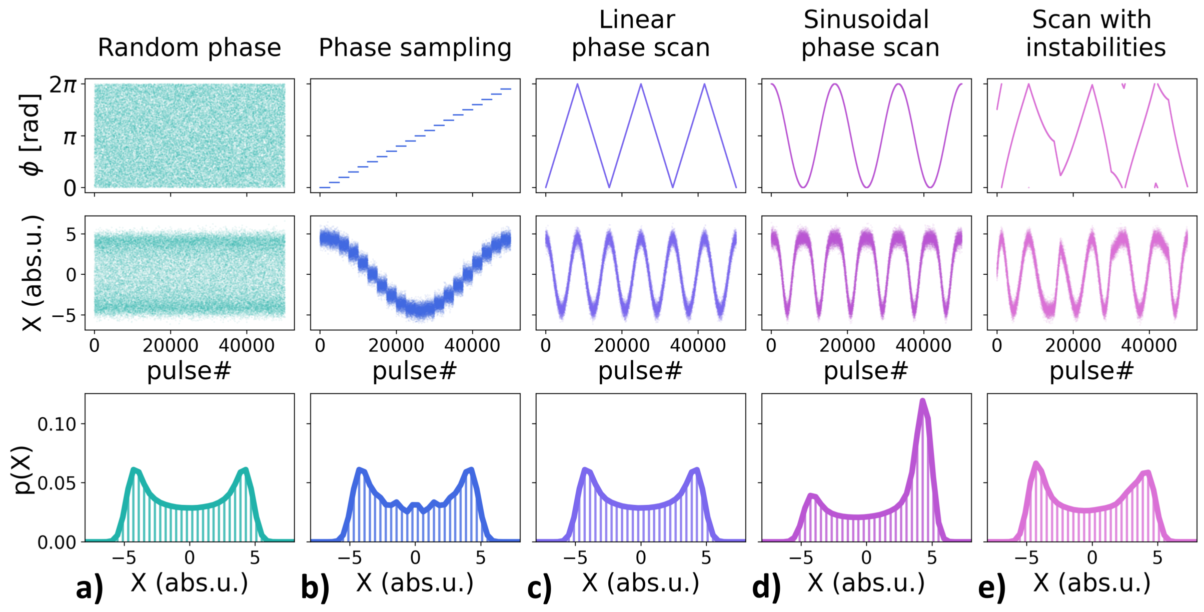

Figure S2: Simulated results for different strategies to implement a phase-averaged detection of coherent state. A specific sequence of phases set at each individual pulse realization (top) produces a set of optical quadrature acquisitions (center) corresponding to different phase-integrated statistical distributions (bottom). The random phase configuration **(a)** and a perfectly linear phase scan **(c)** both produce a perfect phase-averaged distribution. However, finite sampling effects **(b)** or deviations from the ideal linear scan **(d,e)** introduce artifacts in the quadrature distribution.

B Multimode probe characterization

We characterize the multimode spectrum of the ultrashort probe pulse. The frequency-resolved detection is obtained by intereference with a narrow frequency Local Oscillator (bandwidth 0.5 meV), whose spectral content is selected by a pulse shaper [1]. In particular, in our setup we use a Liquid Crystal Spatial Light Modulator (LC-SLM) in a diffraction-based scheme (Fig.S3a). In Fig.S3b we show the photon number distribution obtained as a function of the spectral frequency for some selected frequency modes. We observe as the distribution shifts following the Gaussian profile of the frequency spectrum. From the full distribution we detail the mean value and variance spectra. Mean and variance are superimposed as expected for coherent states with Poissonian statistics.

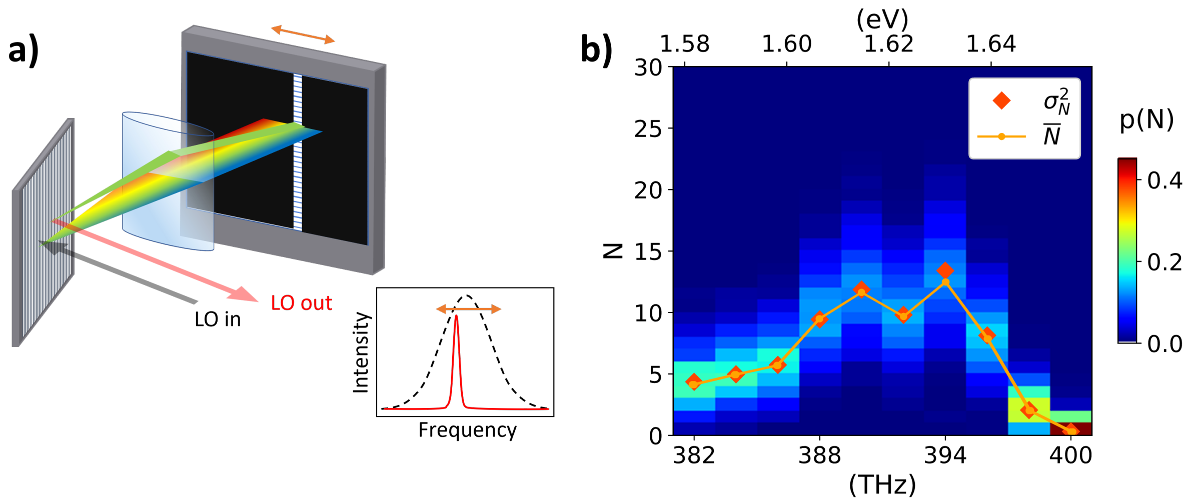

Figure S3: Equilibrium characterization of the multimode probe spectrum. **a)** Diffraction-based pulse shaping scheme. A programmable LC-SLM is adopted to select the Local Oscillator spectral content and implement a frequency-resolved detection. **b)** We report the photon number distribution measured for different probe frequency components. From the full distribution map we calculate the spectra for the mean photon number (orange circles) and the relative variance (red diamonds).

C Quantum shot-noise characterization

Our experimental scheme relies on the rejection of excess noise (i.e. classical experimental fluctuations) which adds to the quantum shot-noise level. In an ideal setting, all the classical fluctuations are perfectly cancelled by the balanced differential detection. However, some residual unbalance is unavoidable, e.g. the one induced by the homodyne interference. In Fig.S4 we characterize the noise as a function of the probe intensity. The classical fluctuations are usually quadratic in the photon number, while the quantum ones are linear. As a consequence of this, we can reduce the relative contribution of classical noise using weak coherent states. From the data in Fig.S4, we can fit the experimental noise with a quadratic trend and compare classical and quantum contributions (${\sigma_N^2}^{(det)} = N + p_2N^2 + p_1N + p_0$). The parameters p_1 and p_0 are negligibly small and we estimate that for a mean photon number < 10 we have a classical to quantum noise ratio $< 2\%$.
The excess noise gives a super-Poissonian statistics and a positive value of the Mandel parameter. Basing on the fit on (a), we can calculate the expected Mandel parameter $Q_{det} = {\sigma_N^2}^{(det)}/N - 1 \simeq p_2N$ and employ it to reference changes in the non-equilibrium dynamics.

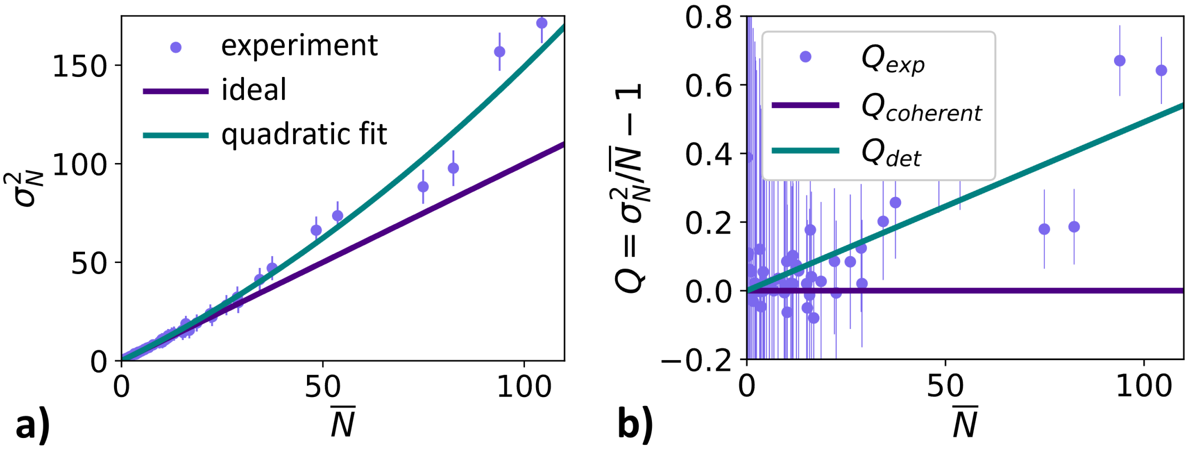

Figure S4: Excess noise characterization at equilibrium as a function of the mean photon number. a) The measurement of the photon number variance progressively deviates from the ideal Poissonian behavior with increasing mean photon number (error bars represent Poissonian noise, 1σ). The contribution of the classical fluctuations is referenced using a quadratic polynomial fit (green line). **b)** Calculation of the Mandel parameter Q. Q_{det} describes the coherent state response taking into account the detection excess noise.

D Quantum model for Impulsive Stimulated Raman Scattering

We study how different phonon statistical properties affect the optical response in a pump-probe experiment using the model presented in the article *Quantum model for Impulsive Stimulated Raman Scattering*, J. Phys. B: At. Mol. Opt. Phys. 52, 145502 (2019) [2], and in chap. 6-7 of F. Glerean's PhD Thesis (https://hdl.handle.net/11368/2988327).

The light-phonon interaction under consideration is a Raman interaction, which can be modeled modeled with an Hamiltonian of the form

$$H_{Ram} = -\sum_{\lambda,\lambda'} \chi^{(1)}_{\lambda,\lambda'} \sum_j \left[\left(a^\dagger_{\lambda j} a_{\lambda' j+\frac{\Omega}{\delta}} \right) b^\dagger + \left(a_{\lambda j} a^\dagger_{\lambda j+\frac{\Omega}{\delta}} \right) b \right] \tag{1}$$

where $a_{\lambda,j}$ and b represent respectively the photon and phonon fields, j labels the frequency component of the multi-mode optical pulse ($\omega = \omega_0 + \delta j$) and λ is the polarization index.

The non-linear polarizability tensor $\chi^{(1)}_{\lambda,\lambda'}$ regulates the strength of the interaction and the symmetry of the phonon mode. We analyze the response of the E^T symmetry mode, which has off-diagonal susceptibility terms, resulting in photon scattering among orthogonal polarizations.

$$H_{Ram} = -\chi^{(1)}_{x,y} \sum_j \left[\left(a^\dagger_{x,j} a_{y,j+\frac{\Omega}{\delta}} \right) b^\dagger + \left(a_{x,j} a^\dagger_{y,j+\frac{\Omega}{\delta}} \right) b + \left(a^\dagger_{y,j} a_{x,j+\frac{\Omega}{\delta}} \right) b^\dagger + \left(a_{y,j} a^\dagger_{x,j+\frac{\Omega}{\delta}} \right) b \right] \tag{2}$$

In the experiment, the probe pulse is mainly polarized along x, and we measure the modulation of the phonon induced scattering in the residual orthogonal component y with a small number of photons per pulse. We calculate the effects of the interaction hamiltonian in the optical field considering the evolution of the operators up to second order in the evolution parameter $\tau\chi^{(1)}$ (τ is the interaction time) as

$$a'_{yj} = a_{yj} + i\tau[H_{Ram}, a_{yj}] - \frac{\tau^2}{2}[H_{Ram}, [H_{Ram}, a_{yj}]] \tag{3}$$

with

$$[H_{Ram}, a_{yj}] = +\chi^{(1)}(a_{xj+\frac{\Omega}{\delta}} b^\dagger + a_{xj-\frac{\Omega}{\delta}} b) \tag{4}$$

and

$$[H_{Ram},[H_{Ram},a_{yj}]] = +(\chi^{(1)})^2\Big(a_{yj+\frac{2\Omega}{\delta}}b^\dagger b^\dagger + a_{yj}b^\dagger b + a_{yj}bb^\dagger + a_{yj-\frac{2\Omega}{\delta}}bb \quad (5)$$

$$+ \sum_j(-a_{xj+\frac{\Omega}{\delta}}a_{xj}a^\dagger_{yj+\frac{\Omega}{\delta}} - a_{xj+\frac{\Omega}{\delta}}a^\dagger_{xj+\frac{\Omega}{\delta}}a_{yj} \quad (6)$$

$$+ a_{xj-\frac{\Omega}{\delta}}a^\dagger_{xj}a_{yj+\frac{\Omega}{\delta}} + a_{xj-\frac{\Omega}{\delta}}a_{xj+\frac{\Omega}{\delta}}a^\dagger_{yj})\Big). \quad (7)$$

The state on which the operators act is a multimode coherent light state combined with a statistical mixture of coherent phonon states. In the density operator formalism we can describe it as

$$\rho = \rho_{light} \otimes \rho_{phonon} = (|\alpha\rangle\langle\alpha|) \otimes (\sum_m p_m |\beta_m\rangle\langle\beta_m|) \quad (8)$$

where $|\alpha\rangle = \otimes_j |\alpha_j\rangle$ (with $a_j|\alpha_j\rangle = \alpha_j|\alpha_j\rangle$) is the multimode state built as tensor product of the individual light modes, while the phonon state is a mixed state describing the statistical ensemble of identical phonon oscillators, where each state $|\beta_m\rangle$ occurs with probability p_m.

Our goal is to study the fluctuations of the system. We can calculate the variance of the generic operator O as

$$\sigma^2_O = \langle O^2\rangle - \langle O\rangle^2 = Tr(\rho O^2) - Tr(\rho O)^2 \quad (9)$$

In order to test the possible results of the experiment we focus on the probe intensity (i.e. the photon number) $N_{yj} = a^\dagger_{yj}a_{yj}$. The probe output operator after the interaction with the sample is precisely

$$N'_{yj} = (a'_{yj})^\dagger a'_{yj} \quad (10)$$

which up to second order explicitely reads

$$N'_{yj} = (a_{yj})^\dagger a_{yj} + i\tau(a^\dagger_{yj}[H,a_{yj}] - [H,a_{yj}]^\dagger a_{yj}) + \quad (11)$$

$$+ \tau^2[H,a_{yj}]^\dagger[H,a_{yj}] \quad (12)$$

$$- \frac{\tau^2}{2}(a^\dagger_{yj}[H,[H,a_{yj}]] + [H,[H,a_{yj}]]^\dagger a_{yj}) \quad (13)$$

from which we can compute

$$\langle N'\rangle = Tr(\rho N') \quad (14)$$

and

$$\langle N'^2\rangle = Tr(\rho N'^2). \quad (15)$$

The expectation value of the probe intensity:

$$\langle N'_{yj}\rangle = \langle(a_{yj})^\dagger a_{yj}\rangle + i\tau\langle a^\dagger_{yj}[H,a_{yj}] - [H,a_{yj}]^\dagger a_{yj}\rangle \quad (16)$$

$$+ \langle\tau^2[H,a_{yj}]^\dagger[H,a_{yj}]\rangle \quad (17)$$

$$- \frac{\tau^2}{2}\langle a^\dagger_{yj}[H,[H,a_{yj}]] + [H,[H,a_{yj}]]^\dagger a_{yj}\rangle \quad (18)$$

at first order can be expressed as

$$\begin{aligned}
\langle N'_{yj}\rangle &= \langle a^\dagger_{yj} a_{yj}\rangle + && (19)\\
&+ \; i\tau\chi\Big(\langle b^\dagger\rangle \left(\langle a^\dagger_{yj}\rangle \langle a_{xj+\frac{\Omega}{\delta}}\rangle + \langle a_{yj}\rangle \langle a^\dagger_{xj-\frac{\Omega}{\delta}}\rangle \right) && (20)\\
&- \; \langle b\rangle \left(\langle a^\dagger_{yj}\rangle \langle a_{xj-\frac{\Omega}{\delta}}\rangle + \langle a_{yj}\rangle \langle a^\dagger_{xj+\frac{\Omega}{\delta}}\rangle \right)\Big) && (21)\\
& && (22)
\end{aligned}$$

The calculation of the squared intensity operator up to second order reads:

$$\begin{aligned}
{N^{2}}'_{yj} &= a^\dagger_{yj} a_{yj} a^\dagger_{yj} a_{yj} + && (23)\\
&+ \; i\tau\Big(a^\dagger_{yj} a_{yj} a^\dagger_{yj} [H, a_{yj}] - a^\dagger_{yj} a_{yj} [H, a_{yj}]^\dagger a_{yj} && (24)\\
&+ \; a^\dagger_{yj} [H, a_{yj}] a^\dagger_{yj} a_{yj} - [H, a_{yj}]^\dagger a_{yj} a^\dagger_{yj} a_{yj}\Big) && (25)\\
&+ \; \tau^2\Big(- a^\dagger_{yj} [H, a_{yj}] a^\dagger_{yj} [H, a_{yj}] + a^\dagger_{yj} [H, a_{yj}][H, a_{yj}]^\dagger a_{yj} && (26)\\
&+ \; [H, a_{yj}]^\dagger a_{yj} a^\dagger_{yj} [H, a_{yj}] - [H, a_{yj}]^\dagger a_{yj} [H, a_{yj}]^\dagger a_{yj} && (27)\\
&+ \; a^\dagger_{yj} a_{yj} [H, a_{yj}]^\dagger [H, a_{yj}] + [H, a_{yj}]^\dagger [H, a_{yj}] a^\dagger_{yj} a_{yj} && (28)\\
&- \; \frac{1}{2}(a^\dagger_{yj} a_{yj} a^\dagger_{yj} [H, [H, a_{yj}]] + a^\dagger_{yj} a_{yj} [H, [H, a_{yj}]]^\dagger a_{yj})\Big) && (29)
\end{aligned}$$

If we use the previous result to calculate the variance, taking into account the commutation relation for $[a_{yj}, a^\dagger_{yj}] = 1$ we obtain

$$\begin{aligned}
\sigma^2_{N'_{yj}} &= \langle {N^{2}}'_{yj}\rangle - \langle N'_{yj}\rangle^2 = \langle {N^{2}}'_{yj}\rangle + && (30)\\
&+ \; \tau^2\Big(- \langle a^\dagger_{yj} a^\dagger_{yj}\rangle \left(\langle [H, a_{yj}][H, a_{yj}]\rangle - \langle [H, a_{yj}]\rangle \langle [H, a_{yj}]\rangle\right) && (31)\\
&+ \; \langle a^\dagger_{yj} a_{yj}\rangle \left(\langle [H, a_{yj}]^\dagger [H, a_{yj}]\rangle - \langle [H, a_{yj}]^\dagger\rangle \langle [H, a_{yj}]\rangle\right) + h.c.\Big) && (32)
\end{aligned}$$

where the first line is the shot noise proportional to the photon number, while the other factors at second order depend on the phonon statistics.

In order to reach a tractable final expression, we underline that we are working with birefringent quartz and that orthogonal modes are out of phase because of the equilibrium refraction which generates elliptical polarization. The ellipticity can be taken into account setting $\phi_{\alpha_x} = 0$ and $\phi_{\alpha_y} = \pi/2$. This makes $\langle a^\dagger_{yj} a^\dagger_{yj}\rangle = |\alpha_{yj}|^2 e^{-i\pi} = -|\alpha_{yj}|^2 = -\langle a^\dagger_{yj} a_{yj}\rangle$, which is crucial to avoid mutual cancellation of additional variance terms.

We explicit the a_{xj} and b fields, taking into account the additional terms resulting from their commutation relations. To simplify and write the final result for the variance in a more compact way, we also neglect the spectral dependence on the light amplitude $\alpha_{xj} = \alpha_x$.

$$\begin{aligned}
\sigma^2_{N'_y} &= \langle {N^{2}}'_{y}\rangle + && (33)\\
&+ \; 4\tau^2\chi^2\alpha_y^2\alpha_x^2\Big(\langle (b^\dagger + b)^2\rangle - \langle b^\dagger + b\rangle^2 \Big) && (34)\\
&+ \; \tau^2\chi^2\alpha_y^2\Big(2(\langle b^\dagger b\rangle - \langle b^\dagger\rangle \langle b\rangle) + 1\Big). && (35)
\end{aligned}$$

To quantify how the quantum statistical response scales when describing a macroscopic field we consider the dependence on the size of an ensemble of M identical and independent probe phonon oscillators, for which we have a total mean phonon number

$$N_{phonons} = M \langle b^\dagger b \rangle . \tag{36}$$

Basing on this, we scale the field operator as $b \longrightarrow \sqrt{M}b$ in the model predictions, which gives

$$\langle N'_y \rangle = \alpha_y^2 + \tau\chi |\alpha_y||\alpha_x| \sqrt{M} \langle b^\dagger + b \rangle \tag{37}$$

and

$$\begin{aligned} \sigma^2_{N'_y} &= \langle N'_y \rangle + & (38)\\ &+ 4\tau^2\chi^2\alpha_y^2\alpha_x^2\Big(M\Big(\langle b^{\dagger 2}\rangle - \langle b^\dagger\rangle^2 + 2(\langle b^\dagger b\rangle - \langle b^\dagger\rangle\langle b\rangle) + \langle b^2\rangle - \langle b\rangle^2\Big) + 1\Big) & (39)\\ &+ \tau^2\chi^2\alpha_y^2\Big(2M(\langle b^\dagger b\rangle - \langle b^\dagger\rangle\langle b\rangle) + 1\Big). & (40) \end{aligned}$$

E Statistical properties of the phonon state

The optical photon number distribution is sensitive to the statistical properties of the phonon state. We study how different phonon states are mapped in the optical observables using numerical simulations [3] of the phonon statistics for some prototypical states of a quantum harmonic oscillator. We present in Fig.S5 the Wigner distribution describing in the phase space the coherent, thermal and squeezed states employed in the simulations. The pump excitation displaces the system from the origin of the phase space. We consider a displacement operator $D(\beta) = \exp(\beta b^\dagger - \beta^* b)$ with $\beta = 2$, The differences between the states are in their statistical phase space distributions. The coherent state has Heisenberg (vacuum) limited uncertainty in both position and momentum ($\sigma_q^2 = \sigma_p^2 = 1/2$). The thermal state has a larger distribution, and we set its width corresponding to a thermal population of 1 phonon. The Bose-Einstein distribution ($\overline{n} = 1/(e^{\hbar\omega/k_B T} - 1)$) predicts an occupation of about 0.7 phonons at 300 K for the 4 THz mode studied in quartz. The squeezed state distributes the minimal Heisenberg uncertainty anisotropically between position and momentum. We considered the action of a squeezing operator $S(\zeta) = \exp[1/2(\zeta^* b^2 - \zeta b^{\dagger 2})]$, with a real squeezing parameter $\zeta = -0.2$. In general, ζ is a complex parameter, the phase of which defines the direction of the squeezing in the phase space, while the amplitude controls the magnitude of the effect. The example in Fig.S5c, with real ζ, models squeezing aligned along the phase space axes. The negative sign results in an increase of the variance at the crest and a decrease at the nodes of the position and momentum operators. A different phase of ζ would shift this redistribution of fluctuations with respect to the phase of the phonon oscillation, modifying the phase but not the amplitude of the changes observed in the phonon statistics. The time-dependent evolution of the phonon is described by a rotation of the state in the phase space around the origin. Formally, we calculate it considering the unitary evolution $U = e^{-iHt}$ ruled by the phonon hamiltonian $H = \Omega b^\dagger b$.

We calculate the optical response inserting the phonon mean displacement and variance in the expression obtained with the model for the Raman interaction. As in the main text, the equilibrium mean value of the probe photons is $N_y = 3$. We set the cross-section parameters $\tau\chi$ so that the product $|\beta|\tau\chi\sqrt{M}$ matches the amplitude of the experimental response.

In Fig.S6 we investigate the quantitative dependence on the simulation parameters. We study the dependence on the probe intensity, phonon amplitude and number of probed phonon

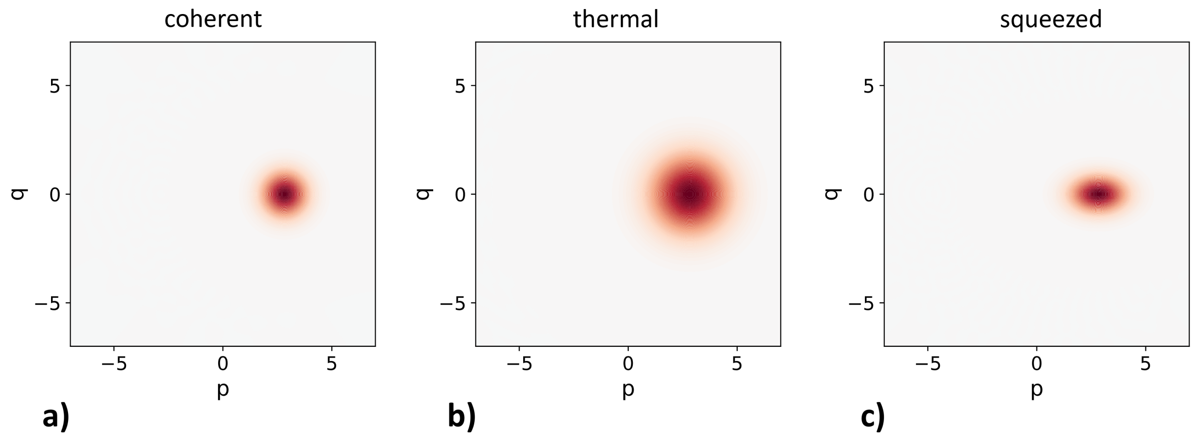

Figure S5: Representation of the phonon state in the phase space. Wigner distributions describing in the phonon position-momentum phase space the coherent **(a)**, thermal **(b)** and squeezed states **(c)** employed in the simulations.

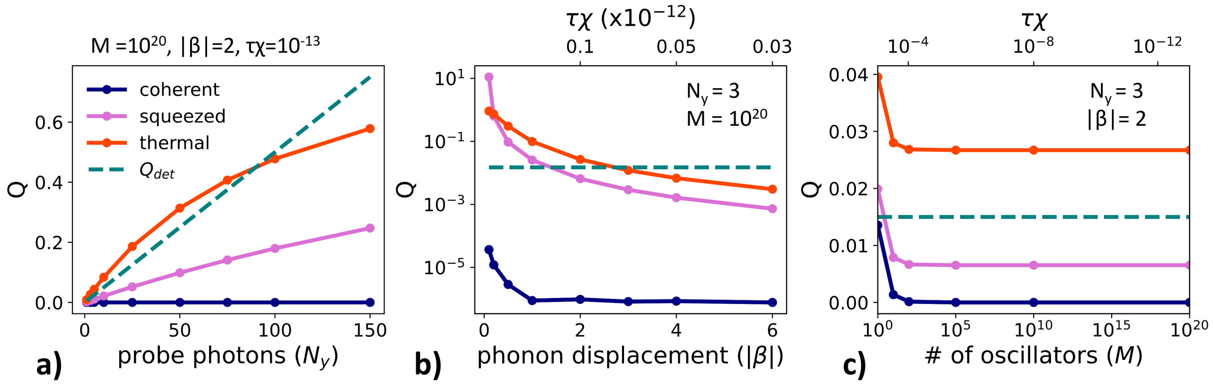

Figure S6: Dependence of the Mandel parameter maximum on the simulation parameters for the different phonon states. a) The deviation from the Poissonian behavior increases with the number of probe photons, but is soon dominated by the increase in the classical detection noise (Q_{det}). **b)** Sets of parameters with the same change in the average photon number give bigger statistical effects for smaller phonon displacements, which correspond to larger non-linear susceptibilities. **c)** The statistical effects are not averaged away when measuring large ensembles of oscillators. Potential quantum effects related to the interaction with the phonon vacuum emerge close to the single system regime.

oscillators. We use as observable the maximum of the optical Mandel parameter over a phonon oscillation. In Fig.S6a we observe that the deviation from the Poissonian behavior increases with the probe intensity, but quickly saturates. For high photon numbers the optical statistics is dominated by the classical detection noise, estimated in appendix C. We understand that although compromising the absolute amplitude of the pump-probe mean-value response, performing the experiment in the few-photon regime is crucial to highlight the contribution of the quantum fluctuations and avoid the one related to the classical noise. In Fig.S6b, we vary $|\beta|$ up to 6 (corresponding to 7000 K for the 4 THz E_T phonon in quartz) keeping the product $|\beta|\tau\chi$ constant. We observe that the modulation of the statistics, measured by Q, decreases as we increase β (i.e. decreasing the cross-section). This indicates that the quantum effects of the light-photon interaction become less important with increasing phonon amplitude or smaller susceptibility.

The results in Fig.S6c show that the amplitude of the statistical effect tend to a well defined value in the limit of a large number of oscillators, without strong dependence on the size of the probed sample. This is important because it reveals that the statistical effects can be accessed

probing macroscopic samples. Interestingly, the Q factor increases for all the phonon distributions close to 1. The latter effect is due to the photon interaction with the phonon vacuum state, which is quantified by the unit factors generated by the commutation relation for the phonon operators $[b, b^\dagger] = 1$. The detection of this latter effect requires though the challenging capability to resolve the response from a microscopic unit.

F Maximum likelihood phase-averaged tomography algorithm

We report on the algorithm employed to perform the phase-averaged tomography procedure. Our goal is to reconstruct the photon number distribution $p(N)$ of the probe state from the measurement of the quadrature X, in particular from the data of its phase-averaged (PHAV) statistical distribution $f(X)$.

The photon number distribution is expressed in terms of the density operator as the diagonal elements of the density matrix in the Fock basis

$$p(N) = \langle N|\,\hat{\rho}\,|N\rangle\,. \tag{41}$$

From the practical point of view, we consider a truncated Fock space with $N < N_{max}$. The numerical limitation of the employed calculator is $N_{max} = 150$.

In order to calculate these terms we use an iterative algorithm which retrieves the density operator which maximizes the probability to obtain the measured data. According to the Maximum Likelihood approach [4, 5], we can retrieve iteratively an estimation of the density operator with the following relation

$$\hat{\rho}^{k+1} = \mathcal{N}[\hat{R}(\hat{\rho}^k)\hat{\rho}^k\hat{R}(\hat{\rho}^k)], \tag{42}$$

where $\mathcal{N}$ accounts for normalization.

The start density operator, $\hat{\rho}^0$, is arbitrary and we set it as the normalized identity operator. The evolution is then obtained calculating the effect of the $\hat{R}$ operator. The $\hat{R}$ operator reads

$$\hat{R}(\hat{\rho}^k) = \int_{-\infty}^{+\infty} dX f(X) \frac{\hat{\Pi}(X)}{\mathrm{Tr}[\hat{\Pi}(X)\hat{\rho}^k]}. \tag{43}$$

The $\hat{\Pi}$ operator describes the measurement process. The phase-resolved homodyne measurement is described by the projector $\hat{\Pi}(\theta, X)$, which is expressed in the Fock basis as

$$\langle m|\,\hat{\Pi}(\theta, X)\,|N\rangle = \langle m|\theta, X\rangle\,\langle \theta, X|N\rangle \tag{44}$$

where

$$\langle N|\theta, X\rangle = \mathrm{e}^{in\theta}\left(\frac{2}{\pi}\right)^{\frac{1}{4}} \frac{H_N(\sqrt{2}X)}{\sqrt{2^N N!}} \exp{(-X^2)}. \tag{45}$$

In our specific case, the PHAV projector reads

$$\hat{\Pi}(X) = \frac{1}{2\pi}\int_0^{2\pi} d\theta\,\hat{\Pi}(\theta, X) \tag{46}$$

for which the previous expressions reduce to

$$\langle m|\,\hat{\Pi}(X)\,|N\rangle = \delta_{m,N}|\,\langle N|X\rangle\,|^2, \tag{47}$$

with

$$|\,\langle N|X\rangle\,|^2 = \left(\frac{2}{\pi}\right)^{\frac{1}{2}} \frac{\big(H_N(X)\big)^2}{2^N N!} \exp{(-\frac{X^2}{2})}^2. \tag{48}$$

when the normalization $X = \frac{1}{\sqrt{2}}(\hat{a} + \hat{a}^\dagger)$ is employed.

The matrix elements of the $\hat{\Pi}(X)$ and $\hat{\rho}^k$ operators are the fundamental blocks used in calculating

the output. The PHAV setting doesn't allow us to reconstruct $\hat{\rho}$ completely, but it is enough to calculate the diagonal elements describing the photon number distribution $\langle N| \hat{\rho}^{k+1} |N\rangle$.
The not-normalized output is expressed by

$$\begin{aligned}\langle N| \hat{\rho}^{k+1} |N\rangle &= \sum_{l,m} \langle N| \hat{R}(\hat{\rho}^k) |l\rangle \langle l| \hat{\rho}^k |m\rangle \langle m| \hat{R}(\hat{\rho}^k) |N\rangle \quad (49)\\ &= \langle N| \hat{\rho}^k |N\rangle \Big(\int_{-\infty}^{+\infty} dX\, f(X) \frac{\langle N| \hat{\Pi}(X) |N\rangle}{\mathrm{Tr}[\hat{\Pi}(X)\hat{\rho}^k]} \Big)^2 \quad (50)\end{aligned}$$

where

$$\mathrm{Tr}[\hat{\Pi}(X)\hat{\rho}^k] = \sum_{l,m} \langle m| \hat{\Pi}(X) |l\rangle \langle l| \hat{\rho}^k |m\rangle = \sum_m |\langle m|X\rangle|^2 \langle m| \hat{\rho}^k |m\rangle\,, \quad (51)$$

in which we used eq. 47.
The calculation is then completed normalizing the output as

$$\langle N| \hat{\rho}^{k+1}_{norm} |N\rangle = \frac{\langle N| \hat{\rho}^{k+1} |N\rangle}{\sum_m \langle m| \hat{\rho}^{k+1} |m\rangle}. \quad (52)$$

The results shown in the present work are obtained running the algorithm for 100 iterations.

References

1. Glerean, F., Jarc, G., Marciniak, A., Giusti, F., Sparapassi, G., Montanaro, A., Rigoni, E. M., Tollerud, J. O. & Fausti, D. Time-resolved multimode heterodyne detection for dissecting coherent states of matter. *Opt. Lett.* **45,** 3498–3501 (2020).
2. Glerean, F., Marcantoni, S., Sparapassi, G., Blason, A., Esposito, M., Benatti, F. & Fausti, D. Quantum model for impulsive stimulated Raman scattering. *Journal of Physics B: Atomic, Molecular and Optical Physics* **52,** 145502 (2019).
3. Johansson, J. R., Nation, P. D. & Nori, F. QuTiP 2: A Python framework for the dynamics of open quantum systems. *Computer Physics Communications* **184,** 1234–1240 (2013).
4. Banaszek, K. Maximum-likelihood estimation of photon-number distribution from homodyne statistics. *Phys. Rev. A* **57,** 5013–5015 (1998).
5. Lvovsky, A. I. Iterative maximum-likelihood reconstruction in quantum homodyne tomography. *Journal of Optics B: Quantum and Semiclassical Optics* **6,** S556–S559 (2004).
